# Is It Proper for Consumptives to Marry?

**Published:** 1871-02

**Authors:** F. A. Hartsen


### IS IT PROPER FOR CONSUMPTIVES TO MARRY ?

BY DR. F. A. HARTSEN.*

This is a complex question. Many, considering the hereditary transmission of phthisis, will answer in the negative. Yet it is certain that successful love, by enlivening all the functions, must contribute to cure the sick, while disappointed affection may readily induce a fatal result, and thus bring misery not only upon the party immediately interested, but also upon others. Now, I ask, is it allowable to imperil the lives of several persons on account of children who may not be born ? There is no law by which the children of consumptives necessarily inherit any pulmonary disease, and, still less, consumption. But, even if the inheritance were inevitable, it should be remembered that death must arrive sooner or later, either by consumption or from some other cause. Now, it cannot be assumed that a consumptive is necessarily less fortunate, less estimable, less talented, or less useful, than other men ; and we therefore cannot perceive any reason why his lot should be rendered more unhappy than his neighbor's. If none but valid men should marry, the world would soon come to a pretty pass. Besides, it is not to be forgotten that the therapeutics of phthisis are improving, and our children will advance them more than we have done. Before the offspring of such marriages become liable to the disease, many a discovery may be made that will render the disease more tolerable and less destructive. Moreover consumptives escape certain dangers to which strong men are exposed,—death in battle, for instance. It is often assumed that sexual relations are especially exhausting to consumptives, and that they are bound to lead a life of total abstinence from carnal indulgence. But this is an exaggeration. Sensible people will enjoy with moderation. Besides, sexual indulgence is less exhausting than certain persons, who have concluded that the grapes are sour, would have us believe. Married people often grow fat, and do not commonly look weak and ailing.

To this plausible argumentation Professor Virchow replies :

Although unused to answer articles published in my *Archives*, I must here make an exception, because the question mooted by Dr. Hartsen demands the most careful judgment, and because even the opponents of truth ought not to be silenced. The answer to the question propounded by him will be diversely answered by a consumptive and by a healthy man. The former, quite independently of the fact that persons of a weak and nervous temperament are prone to sexual pleasures, will reply in the affirmative. He will reason after the fashion of Dr. H., and pos-

*Virchow's Archives, Berlin, 1870.

sibly even go so far as to subordinate the question of marriage and of family to the desire for sexual indulgence. Passion overrides judgment: even a consumptive who is also a physician may not be proof against its delusions. I well remember one of our most accomplished morbid anatomists, who belonged to a tuberculous family, and had, indeed, lost his father and two brothers by consumption. While still a young man he fully resolved never to marry. Nevertheless his time and fate overtook him: he married; and before a year had passed, an acute attack of the disease put an end to his life. As men, let us judge not, lest we be judged; but as physicians, let us not be guided in this matter by consumptives, even although they happen to be physicians. However we may dissuade from sexual indulgence and from marriage, there will be consumptives enough to make light of our judgments. We cannot and we will not proclaim an interdiction, but shall fulfill a moral duty when we raise a warning voice and dissuade from a course of conduct which may become most disastrous to the consumptive, to his family, and to his posterity.

As regards persons of consumptive tendencies, it cannot be doubted that tubercle of the sexual organs of the male (testicles, vas deferens, prostrate,) most commonly occurs about the age of puberty and during the first years after the commencement of sexual relations, although I have occasionally met with it at an earlier age. On the other hand, I have seen consumptives of continent habits whose marriage became the signal for the development of tubercle of the prostrate, etc. A similar fate, though more rarely, overtakes women after their confinement. I have repeatedly seen tuberculous endo-metritis directly developed by the puerperal state; and although this affection is less dangerous than tubercle of the prostrate, yet it readily becomes associated with tuberculous peritonitis, and is therefore not to be made light of. I will not now attempt to decide how far blennorrhœal discharges from the sexual organs promote tuberculous development in them, yet I feel it my duty to remark that, according to my experience, a catarrhal affection may occasion the developement of tubercle in them, just as pulmonary catarrh may induce it in the lungs.

But the question before us relates not only to tuberculosis of the genital organs, but also and mainly to the influence of sexual indulgence in hastening the course of the pulmonary disease or in provoking its relapse. For a long time the deceptive doctrine was preached that pregnancy and childbed exerted a favorable influence upon the course of phthisis, and even upon the tuberculous predisposition. Grisolle and Dubreuilh, however, demonstrated the contrary; and while I, on the strength of numerous observations, am ready to acknowledge that this rule is happily not without exceptions, yet it must be admitted by every experienced physician that parturition involves great danger to tuberculous females. Let it also be considered that not a few women predisposed to consumption refuse, either from sentiment or necessity, to deprive their infants of the maternal food, and that suckling is one of the most deleterious influences among those that determine the fatal downward course of phthisis.

Dr. Hartsen appears to have had more regard for wives than for their husbands. The men are emphatically advised to be prudent and moderate. But what chance has reason against the allurements of sense? A young husband has seldom the opportunity to be moderate, for the danger of marriage consists in the very facility of erring. And in point of fact nothing is more common than to see young men belonging to consumptive families perish in the first years of their marriage. How numerous are the young widows made by such espousals!

But Dr. Hartsen, it appears to me, estimates too lightly the dangers to which the children of consumptive parents are exposed. He rather suggests the hope that the marriage of such persons may be unfruitful. But no man marries expecting to remain childless. He comforts himself with the reflection that all the children of consumptives are not necessarily tuberculous. But in truth there are but few such whose health is not delicate, and who are not in danger at least of leaving to their own children the seeds of the disease, or a predisposition to it. And of these children how large a proportion die of phthisis! However, Dr. H. has at least this faith in the next generation, that it will rapidly advance in its knowledge of curing the disease.

I imagine I have contributed something to inspire this hope, in banishing the ghost of tuberculosis which haunted a goodly number of consumptives, and especially of persons affected with pulmonary phthisis. Many a case of chronic bronchitis, and many of caseous pneumonia, is curable, merely because it is not tuberculous. But it cannot be assumed that all consumptives are tuberculous, and that caseous pneumonia creates no heritable tendency. In my judgment, medicine will never attain to the complete cure of consumption; and therefore it is a sorry consolation to one's self and family to bid them confide in the therapeutics of the future.

Not long ago, and soon after one another, two anxious fathers, in both of whose families consumption had inflicted cruel bereavements, consulted me respecting the proposed marriage of their children. On this, as on other similar occasions, I advised that the young persons should be fully informed of the danger they incurred, and that they should then be allowed to decide the question on their own responsibility. In my opinion, that is the very last limit of concession which a physician ought to yield. In former times lepers were forbidden to marry; but the more humane spirit of our age forbids such constraint. Yet we are as little warranted in advising consumptives to marry, as those who have a hereditary tendency to insanity.

